# Dissociating maternal responses to sad and happy facial expressions of their own child: An fMRI study

**DOI:** 10.1371/journal.pone.0182476

**Published:** 2017-08-14

**Authors:** Dorothea Kluczniok, Catherine Hindi Attar, Jenny Stein, Sina Poppinga, Thomas Fydrich, Charlotte Jaite, Viola Kappel, Romuald Brunner, Sabine C. Herpertz, Katja Boedeker, Felix Bermpohl

**Affiliations:** 1 Department of Psychiatry and Psychotherapy, Charité Campus Mitte, Charité - Universitätsmedizin Berlin, Berlin, Germany; 2 Department of Psychology, Humboldt-Universität zu Berlin, Berlin, Germany; 3 Department of Child and Adolescent Psychiatry, Psychosomatics and Psychotherapy, Charité Campus Virchow, Charité - Universitätsmedizin Berlin, Berlin, Germany; 4 Department for General Psychiatry, Center of Psychosocial Medicine, University of Heidelberg, Heidelberg, Germany; 5 Section for Disorders of Personality Development, Clinic of Child and Adolescent Psychiatry, Centre of Psychosocial Medicine, University of Heidelberg, Heidelberg, Germany; Universita degli Studi di Udine, ITALY

## Abstract

**Background:**

Maternal sensitive behavior depends on recognizing one’s own child’s affective states. The present study investigated distinct and overlapping neural responses of mothers to sad and happy facial expressions of their own child (in comparison to facial expressions of an unfamiliar child).

**Methods:**

We used functional MRI to measure dissociable and overlapping activation patterns in 27 healthy mothers in response to happy, neutral and sad facial expressions of their own school-aged child and a gender- and age-matched unfamiliar child. To investigate differential activation to sad compared to happy faces of one’s own child, we used interaction contrasts. During the scan, mothers had to indicate the affect of the presented face. After scanning, they were asked to rate the perceived emotional arousal and valence levels for each face using a 7-point Likert-scale (adapted SAM version).

**Results:**

While viewing their own child’s sad faces, mothers showed activation in the amygdala and anterior cingulate cortex whereas happy facial expressions of the own child elicited activation in the hippocampus. Conjoint activation in response to one’s own child happy and sad expressions was found in the insula and the superior temporal gyrus.

**Conclusions:**

Maternal brain activations differed depending on the child’s affective state. Sad faces of the own child activated areas commonly associated with a threat detection network, whereas happy faces activated reward related brain areas. Overlapping activation was found in empathy related networks. These distinct neural activation patterns might facilitate sensitive maternal behavior.

## Introduction

The parent-child relationship is important for the development of emotional and social skills in early childhood. Sensitive parental behavior has been argued to be essential for the normal development and mental health of a child [1–3]. Beside physical contact, emotional signalizing (i.e., revealing one’s own emotional state) and the recognition of such signals are characteristic of high parental sensitivity [4]. Distinguishing different emotional states of one’s own child is an important ability in order to fulfill the child’s needs and to validate his feelings. In the following paper, we will refer to this ability as maternal affect recognition. In addition, we will focus on maternal affect recognition, even though we assume that paternal affect recognition (or that of significant other caregivers) would be of similar importance for a child’s development. If we better understand the neural basis of maternal affect recognition, we might be able to better understand (and potentially improve) sensitive, as well as neglectful maternal parenting behavior [5,6].

A number of fMRI studies have investigated maternal brain responses to images of their own child’s face (for a review, see [7–9]). Viewing the face of one's own compared to that of another child consistently activated brain areas involved in reward (e.g., ventral striatum, hippocampus), threat detection (e.g., amygdala, anterior cingulated cortext), and empathy processes (e.g., inferior frontal/orbito-frontal cortex, insula) [10–14]. On a behavioral level, brain activation in these areas was associated with maternal valence and arousal ratings (e.g.,[11, 15]), possibly indicating maternal attachment to her child. While the majority of these previous studies mainly focused on maternal responses to neutral and happy facial expressions of their own child, fewer studies included sad facial expressions [13–17]. Since handling of both affects might of the same importance for the mother-child interaction, the question arises, whether maternal responses to sad differ from responses to happy faces of their own child.

In addition, these previous studies have focused on maternal responses to facial expressions of infants (0 to 3 years of age), while studies on older children are lacking. To the best of our knowledge only one study has previously investigated in a small sample of mothers (n = 7) their neural response to faces of their school-aged (five to 12 years of age) children [18]. Even though sensitive parenting remains important for infants and children alike, the demands on a healthy mother-child interaction might change, when children move from infancy to school age. School-aged children begin to develop a more complex understanding of emotions and to experience more complex and even “mixed” emotions [19, 20]. This might impact on maternal affect recognition skills, as the emotional signals of her child become more subtle and involve a broader variety of affective states. To account for different child affective states, we implemented an affect recognition task with happy, neutral, and sad facial expressions that allowed us to examine overlapping and distinct neural activation patterns of mothers in response to these different child affective states.

In the present study of healthy mothers, we created individual picture stimuli for each mother, depicting the own and an unfamiliar child with neutral, happy, and sad facial expressions, respectively. Children were between five and 12 years old. During fMRI, mothers were presented with the individual pictures and performed an affect recognition task on the facial expressions. By investigating dissociable and overlapping neural responses to happy and sad facial expressions of one’s own child (compared to an unfamiliar child) we sought to gain further insight into the neural underpinnings of personally relevant emotional stimuli in healthy mothers of school-aged children.

We predicted that recognizing one’s own sad child (OC) compared to an unfamiliar child (UC) would be associated with activation of areas involved in threat and salience detection (e.g., amygdala), whereas recognizing one’s own happy child compared to an unfamiliar happy child would be mainly associated with activation of areas involved in reward processing (e.g., ventral striatum, hippocampus). Conjoint activation in response to happy and sad facial expressions of one’s child should be seen in areas associated with empathy (e.g., insula). This neural activation pattern should be further associated with the behavioral post-scan valence and arousal ratings of child’s facial expressions. We predicted that positively valenced and highly arousing child pictures would be associated with greater activation in reward related regions (i.e., ventral striatum, hippocampus), whereas negatively valenced and highly arousing child pictures would be associated with greater activation in areas related to salience detection (i.e., amygdala).

## Methods

### Subjects

Thirty healthy subjects were recruited by advertisement. Subjects were right-handed women between 27 to 48 years of age (mean: 39.5 ±5.5) whose (nonadopted) healthy child was between five and 12 years old (mean: 7.8 ±1.6; 43% girls). Handedness was assessed using the Edinburgh Handedness Inventory [21]. The absence of psychiatric illness was confirmed by a structured psychiatric diagnostic interview for axis-I disorders (M.I.N.I; Mini international neuropsychiatric interview; [22]) and axis-II disorders (for the following three personality disorders: emotional-instable, unsecure, antisocial; International Personality Disorder Examination; [23]). The study was approved by the ethics committee of the Charité - Universitätsmedizin, Berlin. The present study was performed within the framework of the project UBICA (“Understanding and Breaking the Intergenerational Cycle of Abuse”). Findings from the clinical samples of the UBICA study will be reported elsewhere. We here focus on findings from healthy subjects recruited at the Berlin study site. All subjects gave written informed consent before participating. Subjects were reimbursed for their participation. Data of three subjects were excluded from analyses because of excessive head movement (exclusion criterion > 2 mm and/or 2° within one run), leaving twenty-seven subjects for final analyses.

### Pictures

In preparation for the fMRI experiment, individual picture stimuli were created for each mother which depicted sad, happy, and neutral facial expressions of her child. These pictures were obtained during a mood induction session with a trained study assistant: First, a mindfulness exercise was performed first, to make children more aware of their external surrounding as well as their internal body sensations. Children were then asked to remember a sad situation they had experienced recently. They were asked to look, walk and behave as if they were sad again. Thereafter, children watched short sad video clips of movies (e.g., “The Lion King” by Disney) while their facial expressions were videotaped. Happy expressions were obtained while children watched comic movies (e.g., “Mickey Mouse” by Disney). Neutral expressions were obtained prior to mood induction. Pictures were extracted by screenshots from the video clips and transformed to grey scale. A team of three study associates rated all facial pictures with respect to the shown valence on a 7-point Likert-scale ranging from very sad (-3) to very happy (+3). Pictures were selected if the rater team agreed upon the valence. Only pictures with moderate valence (i.e., a score of +2 for happy and a score of -2 for sad) and neutral pictures (i.e., a score of 0) were selected for the experiment ensuring a perfect match between the own and unfamiliar child. This valence category was chosen because this might best reflect the everyday task of mothers to correctly recognize their child’s affective state which might often be subtle. In cases where the rater team agreed on more than 30 pictures of a valence, 30 pictures were randomly selected. In cases where raters agreed on less than 30 pictures, pictures were selected when at least two out of three raters agreed. Pictures of six children (three boys and three girls, aged six, eight, and ten years) were used as control stimuli (unfamiliar child, UC). Control children were matched for sex and age (i.e., a boy of similar age was selected for mothers of boys). We admit, however, that we did not match the control pictures for physical similarity (e.g., eye colour, face shape). However, in all pictures, the children faced the camera straight on. Pictures were digitized and cropped to show only the faces.

### Task

During fMRI, pictures of the own and the matched unfamiliar child were presented to the mother who performed an affect recognition task. While viewing the pictures, mothers were asked to indicate via button press for each facial expression as soon and as accurately as possible whether it was sad, happy, or neutral. With this well-established affect recognition task we were able to ensure a behavioral feedback from mothers that they actively looked at the pictures. We used a 3x2 factorial design, comprising the factor “child affect” (neutral, happy, sad) and “identity” (own vs. unfamiliar). Mothers saw 90 pictures of their own child (OC) and 90 of an unfamiliar control child (UC), with 30 pictures for each facial affect, respectively. Pictures were presented in random order for two seconds, followed by a variable inter-trial interval of two to six seconds where a fixation cross was presented. The paradigm consisted of two runs (9 min each). Pictures were presented to the mother on an overhead mirror display. Before entering the scanner, subjects performed a training session to get familiarized with the task. After scanning, mothers were asked to rate each picture in terms of valence and arousal on a 7-point scale using an adapted version of the Self-Assessment Manikin (SAM; [24]).

### Imaging

MRI data were acquired on a 3 Tesla whole-body MRI scanner (MAGNETOM Trio, TIM-Technology; Siemens, Erlangen, Germany) with a 32-channel head coil and a standard T2-weighted echo planar imaging (EPI) sequence, sequential descending acquisition, flip angle α = 78°, 64x64 pixels in-plane resolution, 33 slices, voxel dimensions 3x3x3 mm^3^, a 0.75-mm gap between slides, field of view 192x192 mm^2^. For each run, 257 scans were acquired whereas the first six images were discarded to account for possibly incomplete signal saturation. After the functional session, a T1-weighted high-resolution structural scan was obtained to detect potential brain abnormalities (none was detected).

### Statistics

#### Behavioral data

Response times, hit rates (i.e., percentage of correct answers for each condition), post-scan valence and arousal ratings for the six conditions were compared using separate repeated-measures analyses of variance (ANOVA) with child affect (sad, happy, neutral) and identity (own vs. unfamiliar) as within-subject factors. Degrees of freedom were Greenhouse-Geisser corrected whenever necessary [25]. For clarity, the uncorrected degrees of freedom are reported. Significance was set at p<0.05. Post hoc analyses were performed using paired t-tests with Bonferroni correction for multiple comparisons (p = 0.05/6 = 0.008). Response times and hit rates were missing for one subject due to technical problems. All behavioral data analyses were carried out using SPSS 18 (IBM; Armonk, NY, USA). Possible associations between mothers’ self-reported arousal and valence ratings for one’s own child and peak activation of BOLD responses in predetermined ROIs (see below) were examined in correlational analyses.

#### fMRI analysis

Data analysis was performed with Statistical Parametric Mapping 8 (SPM 8, Wellcome Department of Cognitive Neurology, London, UK) running on MATLAB (Mathworks Inc, Sherborn, MA, USA), with the following preprocessing procedure: slice-time correction and spatial realignment, normalization to the standard Montreal Neurological Institute (MNI) echo-planar imaging (EPI) template using third-degree B-spline interpolation and spatial smoothing with an 8-mm full width at half maximum isotropic Gaussian kernel. Incorrect trials were unequally distributed across conditions and between subjects. We thus did not exclude the relatively low number of incorrect trials from the analyses to provide comparable beta estimates. This approach was also chosen because (1) BOLD responses to emotionally valenced faces can occur even if the subject is not aware of the affective content of the face [26, 27]and (2) we assumed that misinterpreting the valence of a face stimulus would potentially blur the differences between valence conditions, but not cause false positive findings. Statistical analysis was carried out using the first-level general linear model (GLM) function in SPM. For each subject, six experimental conditions (child facial affect: neutral, happy, or sad expressions; identity: own or unfamiliar child) were used as regressors (3x2 factorial design). Hemodynamic responses to each stimulus were modeled with a delta function convolved with a synthetic hemodynamic response function. Low-frequency noise was removed by applying a highpass filter (cut-off: 128 s) to the fMRI time-series at each voxel. Linear contrasts for each experimental condition were created by averaging the same experimental conditions across the two runs. These individual contrasts were submitted to the group level using a second-level full-factorial analysis. To investigate differential activation to sad and happy facial expressions, interaction analyses were done. Specifically, for sad valence*identity, the following interaction contrasts were evaluated:

1) ((sad__OC_ > neutral__OC_) > (sad__UC_ > neutral__UC_))
2) ((sad__OC_>happy__OC_) > (sad__UC_>happy__UC_)).

Likewise, the following interaction contrasts were evaluated for happy valence * identity:

3) ((happy__OC_ > neutral__OC_) > (happy__UC_ > neutral__UC_))
4) ((happy__OC_ > sad__OC_) > (happy__UC_ > sad__UC_)).

For all brain areas, we used a statistical threshold of p<0.05, corrected for multiple comparisons across the whole brain (family-wise error (FWE)). Given our *a priori* interest in responses to sad and happy facial expression, we defined the following areas of interest: amygdala as an area associated with salience and threat detection (hypothesized to activate to sad faces), ventral striatum and hippocampus as areas associated with reward processes (hypothesized to activate to happy faces), and the insula as an are associated with empathy processes (hypothesized to activate to both sad and happy faces).

The whole brain analyses were followed by region-of-interest (ROI) analyses in above mentioned regions if the whole brain analysis did not yield a significant result. Bilateral masks were obtained from the SPM AAL atlas (Automated Anatomical Labeling) provided by wfu pickatlas toolbox [28]. As the AAL atlas does not provide a mask for the ventral striatum, the central coordinates of the ventral striatum (x = ±14, y = 8, z = -8) were taken from an fMRI study targeting reward processing [29]. A small volume correction (SVC) was done based on an 8 mm sphere centered on this voxel. Coordinates are reported in MNI (Montreal Neurological Institute) space. Designations of anatomical regions were based on the canonical T1-image provided by SPM and regions were identified using the WFU Pickatlas [28] and confirmed manually using a human brain atlas [30]. The statistical threshold was set at p<0.05 (FWE corrected). To control for the effect of children’s age we calculated correlations between children’s age and the peak voxel BOLD responses of the above mentioned regions of interests (Spearman rank coefficient).

Finally, we conducted a conjunction analysis (conjunction null hypothesis method) based on the full-factorial analysis for the interaction contrasts ((sad__OC_ > neutral__OC_) > (sad__UC_ > neutral__UC_)) and ((happy__OC_ > neutral__OC_) > (happy__UC_ > neutral__UC_)) to determine significant common activation patterns underlying both, happy and sad faces of the own child compared to a gender- and age-matched unfamiliar child (p<0.05, FWE corrected).

## Results

### Behavioral data

#### Response times

Mean response times and hit rates are shown in Table 1. They were comparable to response times reported by Leveroni and colleagues [13]. We found a significant main effect for valence category (F_(2,50)_ = 35.459, p≤.001; η^2^ = .59) with the lowest response times for happy faces (all p-values≤.001). The main effect for identity failed to reach significance (F_(1,25)_ = 3.742, p>.10; η^2^ = .13). The valence by identity interaction was significant (F_(2,50)_ = 6.919, p≤.01; η^2^ = .22) showing that mothers recognized happy faces of their own child faster than happy faces of the unfamiliar child (t_(25)_ = -6.928; p≤.001).

**Table 1:** Behavioral data across picture conditions.

Picture condition	Response times^a^		Hit rates^b^		Valence ratings^c^		Arousal ratings^d^	
	Mean	SD	Mean	SD	Mean	SD	Mean	SD
OC neutral	1118.3	162.2	78.8	15.8	4.1	0.6	4.1	0.7
OC happy	808.0	102.8	99.9	0.7	6.6	0.4	5.8	1.1
OC sad	1008.2	191.8	90.6	12.3	2.5	0.6	5.1	1.0
UC neutral	1057.8	156.6	84.1	15.5	4.0	0.4	3.7	0.8
UC happy	877.0	82.4	91.0	12.3	5.9	0.6	4.8	1.1
UC sad	1081.7	243.0	87.3	13.1	2.9	0.6	4.2	1.1

OC: own child; UC: unfamiliar child; SD: standard deviation

^a^ in ms

^b^ percentage of correct answers

^c^ rated 1–7, with 1 being most negative and 7 being most positive

^d^ rated 1–7, with 1 being least arousing and 7 being most arousing

#### Hit rates

The main effect of valence category was significant (F_(2,50)_ = 23.481, p≤.001; η^2^ = .48) with the highest accuracy for happy faces (all p-values≤.001). The main effect of identity (F_(1,25)_ = 0.000, p = 1.00; η^2^ = .00) and the valence by identity interaction (F_(2,50)_ = 2.424, p>.05; η^2^ = .09) did not reach significance.

#### Valence ratings

Mean valence and arousal ratings across the six picture conditions are shown in Table 1. We found a significant main effect of valence category (F_(2,52)_ = 409.810, p≤.001; η^2^ = .94) with the highest valence ratings for happy and the lowest for sad faces (all p-values≤.001). The main effect of identity did not reach significance (F_(1,26)_ = 2.797, p < .10; η^2^ = .10). The valence by identity interaction was significant (F_(2,52)_ = 16.279, p≤.001; η^2^ = .39): post-hoc tests revealed that maternal valence ratings were more negative for the own child compared to the unfamiliar child in the sad (mean difference = - .38; SD = .63; t_(26)_ = -3.168, p < .01) compared to neutral condition (mean difference = .00; SD = .71; t_(26)_ = -.027, p>.10). Similar results were found for happy faces: mothers rated faces of their own child more positive than of the unfamiliar child in the happy condition (mean difference = .70; SD = .62; t_(26)_ = 5.786, p < .001) compared to the neutral condition (mean difference = .00; SD = .71; t_(26)_ = -.027, p>.10).

#### Arousal ratings

There were significant main effects of valence category (F_(2,52)_ = 37.722, p≤.001; η^2^ = .60) and identity (F_(1,26)_ = 35.641, p≤.001; η^2^ = .58) with the lowest arousal ratings for neutral faces, highest arousal ratings for happy faces, and higher ratings for the own child (all p-values≤.001). We found a significant valence by identity interaction (F_(2,52)_ = 7.489, p≤.001; η^2^ = .22). Post-hoc tests revealed that maternal arousal ratings were higher for the own child compared to the unfamiliar child in the sad (mean difference = .85; SD = .96; t_(26)_ = 4.582, p < .001) compared to neutral condition (mean difference = .41; SD = .60; t_(26)_ = 3.645, p < .001). Similar results were found for happy faces: maternal arousal ratings were higher for the own child compared to the unfamiliar child in the happy (mean difference = 1.0; SD = .77; t_(26)_ = 6.552, p < .001) compared to neutral condition (mean difference = .41; SD = .60; t_(26)_ = 3.645, p < .001).

### Neuroimaging data

#### sad valence * identity interaction

**Fig 1:**
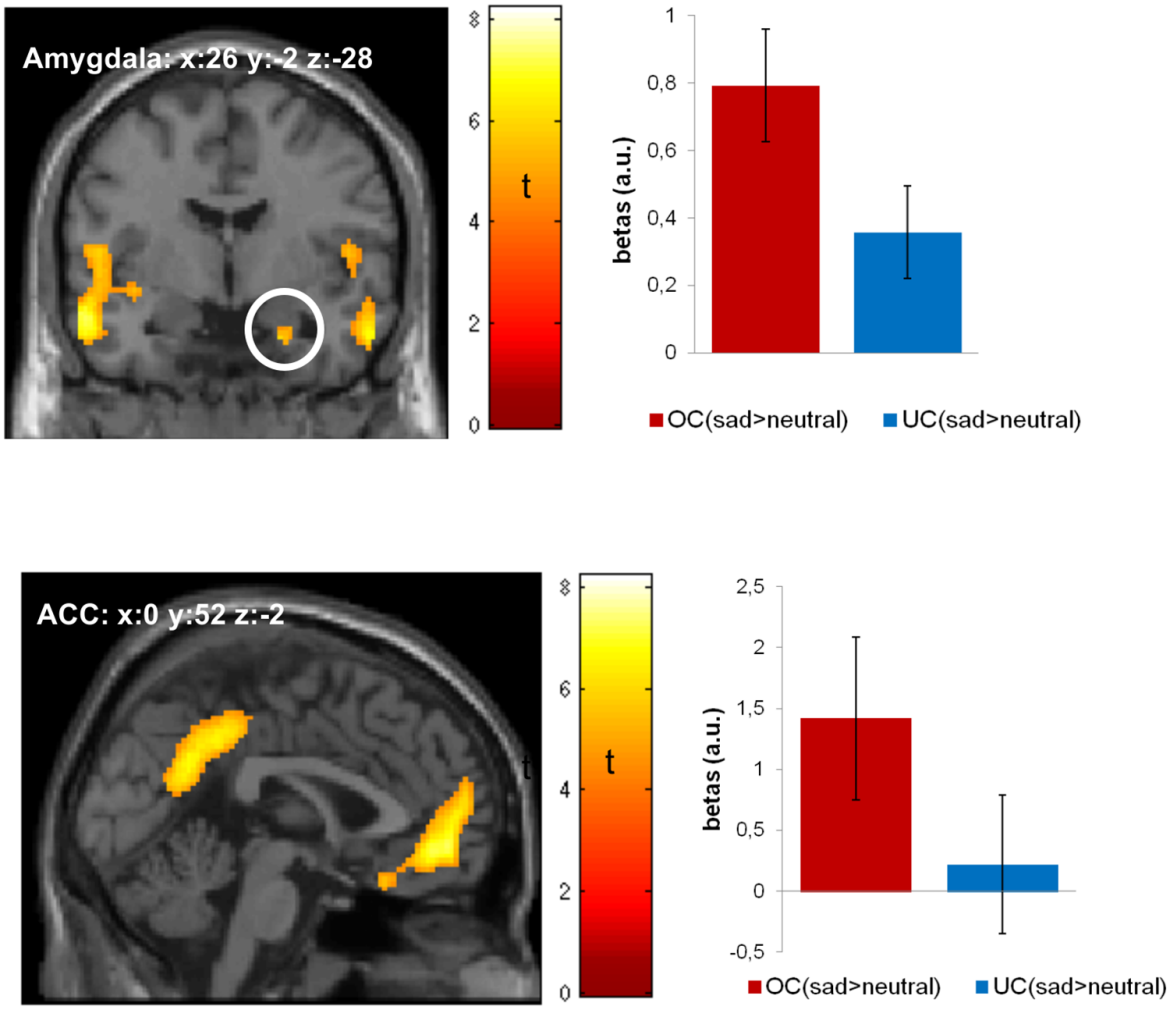
Activation maps for the contrast ((sad_OC > neutral_OC) > (sad_UC > neutral_UC)) are thresholded at p<0.05 (FWE corrected) and overlayed on a standard MRI template; bar plots represent the peak voxel parameter estimates, error bars represent one standard error of the mean; a.u.: arbitrary units; OC: own child; UC: unfamiliar child; ACC: anterior cingulate cortex.

In a first step, we studied the effect of sad facial expressions (of the own child) by contrasting the effect of sad versus neutral valence in pictures of the own (versus unfamiliar) child. The whole brain analysis (p < .05, FWE corrected) revealed significant activations in the anterior cingulate cortex, hippocampus, orbito-frontal gyrus, and insula (Table 2: Fig 1). Further significant activation was observed in the amygdala (ROI, p < .05, FWE corrected (Table 2; Fig 1). None of these activation patterns in response to sad valence by identity interaction was correlated with children’s age (all ps>.254). There were no significant correlations between maternal behavioral ratings and activation in the predetermined ROIs.

**Table 2:** Results for the contrast: (sad_oc > neutral_oc) > (sad_uc > neutral_uc).

Region	BA	R/L	MNI coordinates			T value
			x	y	z	
Frontal						
Superior frontal gyrus^a^	9	L	-20	24	42	5.87
Middle orbitofrontal gyrus^a^	10	R	2	46	-8	6.98
Anterior cingulate gyrus^a^	32	L	-8	30	-8	5.69
	32	R	0	52	-2	6.48
Precentral gyrus^a^	44	R	50	0	6	5.66
Temporal						
Posterior superior temporal gyrus^a^	39	L	-54	-56	8	5.07
Middle temporal gyrus^a^	21	L	-58	-10	-22	8.22
Putamen^a^	21	R	58	-10	-22	6.50
Parietal						
Posterior cingulate/Precuneus^a^	31	L	-2	-54	28	6.64
Inferior parietal gyrus^a^	40	L	-62	-30	26	5.74
	40	R	52	-30	26	5.74
Postcentral gyrus^a^	3	R	38	-34	68	6.10
Occipital						
Cuneus^a^	19	L	-12	-90	24	5.04
	18	R	6	-84	22	5.19
Middle occipital gyrus^a^	39	R	48	-66	24	5.07
Subcortical						
Amygdala^b^		L	-28	-4	-24	4.03
		R	26	-2	-28	4.56
Hippocampus^a^		L	-26	-10	-24	5.80
		R	28	-14	-22	5.52
Parahippocampal gyrus^a^	36	L	-24	-30	-18	4.78
	35	R	22	-4	-28	5.72
Insula^a^	13	L	-38	-6	-8	5.42
	13	R	36	4	8	5.13

BA: Brodman’s area; R: right, L: left; MNI: Montreal Neurological Institute;

^a^ p < .05 (FWE), corrected for whole-brain volume;

^b^p < .05 (FWE), corrected for small volume (SVC)

In a second step, we examined dissociable effects of sad versus happy facial expressions. For this purpose, we contrasted the effect of sad versus happy valence in pictures of the own (versus unfamiliar) child. The whole-brain analysis (p < .05, FWE corrected) indicated almost identical results as in the first interaction term involving neutral faces, i.e., effects in the anterior cingulate cortex, hippocampus, orbito-frontal gyrus, amygdala, and insula (S1 Table).

#### happy valence * identity interaction

**Fig 2:**
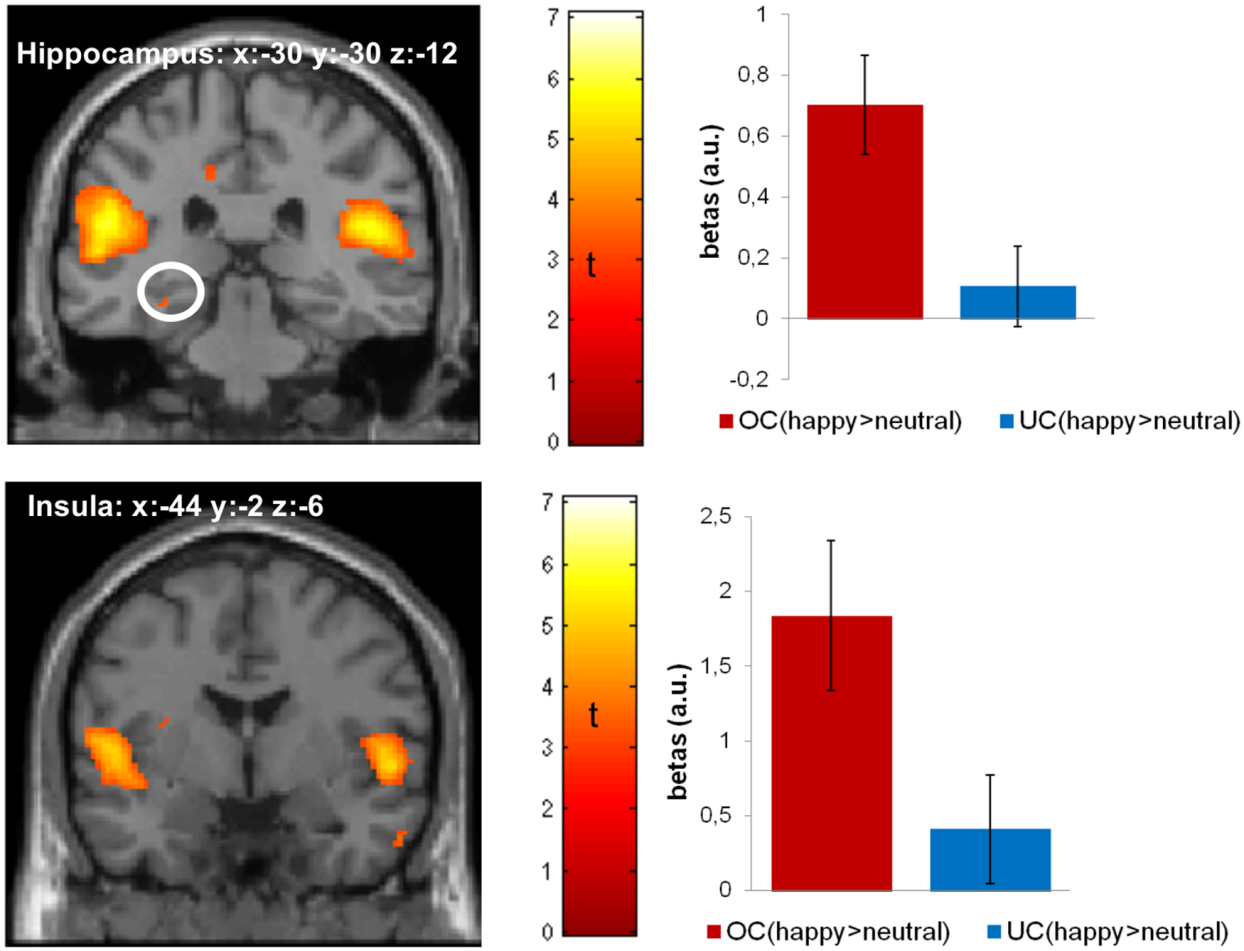
Activation map for the contrast ((happy_OC > neutral_OC) > (happy_UC > neutral_UC)) is thresholded at p<0.05 (FWE corrected) and overlayed on a standard MRI template; bar plots represent the peak voxel parameter estimates, error bars represent one standard error of the mean; a.u.: arbitrary units; OC: own child; UC: unfamiliar child.

Similar to our analyses for sad faces we first studied the effect of happy facial expressions (of the own child) by contrasting the effect of happy versus neutral valence in pictures of the own (versus unfamiliar) child. The whole-brain analysis (p < .05, FWE corrected) for the happy valence * identity effect revealed significant activations in the right parahippocampal region, superior temporal gyrus, inferior parietal gyrus, and the right cuneus (Table 3). The ROI analyses indicated significant activations in the left hippocampus and bilateral insula (p < .05, FWE corrected) (Table 3; Fig 2). As for sad faces, none of these activation patterns were correlated with maternal behavioral ratings nor children’s ages (all ps>.184).

**Table 3:** Results for the contrast: (happy_oc > neutral_oc) > (happy_uc > neutral_uc).

Region	BA	R/L	MNI coordinates			T value
			x	y	z	
Temporal						
Superior temporal gyrus^a^	22	L	-50	-4	4	4.87
	41	R	50	-30	16	5.57
Parietal						
Inferior parietal gyrus^a^	41	L	-50	-34	22	6.43
Occipital						
Middle occipito-temporal gyrus^a^	19	L	-16	-52	-8	5.31
Cuneus^a^	19	R	6	-84	26	7.07
Subcortical						
Insula^b^	13	L	-44	-2	-6	4.16
	13	R	38	-14	14	4.38
Hippocampus^b^		L	-30	-30	-12	3.37
Parahippocampal gyrus^a^	36	R	20	-44	-4	4.33

BA: Brodman’s area; R: right, L: left; MNI: Montreal Neurological Institute;

^a^p < .05 (FWE), corrected for whole-brain volume;

^b^p < .05 (FWE), corrected for small volume (SVC);

Our behavioral data indicated that happy facial expressions were recognized faster and rated as more arousing. To rule out that our results were confounded by a better perceptibility of happy facial expressions, we performed an additional second-level full factorial analysis with reaction times and arousal ratings as covariates. Similar to the initial analysis, this one indicated activation in the right parahippocampus and inferior parietal gyrus (on an uncorrected significance level of p < .001). In addition, activation in the inferior frontal gyrus (p < .001, uncorrected) was found.

In a second step, we examined dissociable effects of happy versus sad facial expressions (of the own child). For this purpose, we contrasted the effect of happy versus sad valence in pictures of the own (versus unfamiliar) child. The whole-brain analysis (p < .05, FWE corrected) indicated significant activation in the inferior frontal gyrus (S2 Table). Further ROI analyses (p < .05, FWE corrected) did not indicate significant results.

For completion, we also explored possible effects of the unfamiliar versus the own child (unfamiliar child > own child) which did not yield any significant activation patterns (p < .05, FWE corrected).

#### Conjunction analysis

**Fig 3:**
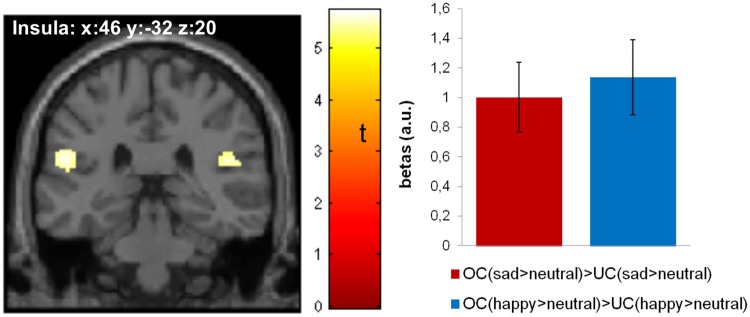
Activation map for the conjunction analysis is thresholded at p<0.05 (FWE corrected) and overlayed on a standard MRI template; bar plots represent the peak voxel parameter estimates, error bars represent one standard error of the mean; a.u.: arbitrary units; OC: own child; UC: unfamiliar child.

A conjunction analysis was carried out based on the interaction contrasts ((happy__OC_ > neutral__OC_) > (happy__UC_ > neutral__UC_)) and (sad__OC_ > neutral__OC_) > (sad__UC_ > neutral__UC_) to identify common neural responses to the affect of the own child, either sad or happy. The whole brain analysis (p < .05, FWE corrected) showed overlapping effects in the superior temporal gyrus, cuneus, and insula (Table 4; Fig 3). The ROI analysis (p < .05, FWE corrected) yielded significant activation in the left hippocampus.

**Table 4:** Results for the conjunction analysis.

Region	BA	R/L	MNI coordinates			T value
			x	y	z	
Temporal						
Superior temporal gyrus^a^	40	L	-52	-34	20	5.73
Occipital						
Cuneus^a^	18	R	6	-84	22	5.19
Subcortical						
Insula^a^	22	L	-50	-4	4	4.87
	13	R	46	-32	20	5.54
Hippocampus^b^		L	-30	-30	-12	3.37

BA: Brodman’s area; R: right, L: left; MNI: Montreal Neurological Institute;

^a^p < .05 (FWE), corrected for whole-brain volume;

^b^p < .05 (FWE), corrected for small volume (SVC);

## Conclusions

The aim of the present study was to examine dissociable and overlapping activation patterns in mothers viewing sad and happy faces of their own (compared to an unfamiliar) child. Our paradigm is one of the first that allows one to directly compare sad and happy faces of the own child. We found that viewing one’s own sad child was associated with differential activation in the amygdala, anterior cingulate cortex, insula, and hippocampus, which may be considered areas of a threat detection network. In contrast, viewing one’s own happy child did not activate the amygdala and anterior cingulate cortex, but the (para)hippocampus and insula, i.e., areas which may be considered part of a reward network. Overlapping activations for sad and happy faces of the own child included the insula and superior temporal gyrus, which may relate to an empathy network. Our findings suggest that affect recognition of sad and happy facial expressions of the own child activates overlapping and dissociable networks, which might lay the foundations for sensitive maternal behavior with differential maternal responses to distinct child emotions.

Our present study adds to the existing literature in that (1) beside happy and neutral expressions, also sad faces were presented (in comparison to [18]), (2) our data suggest that dissociable responses to sad and happy facial expressions can already be observed during perceptive stages of the maternal responses, even during early subcortical visual information processing (in line with literature using auditory stimuli (e.g., [32, 33])), and (3) we investigated maternal neural responses to their school-aged child (as opposed to e.g., [14, 15]).

Most previous studies on maternal perception of the own child’s face have relied on neutral and happy expressions [10–12; 34] and found activation in the orbitofrontal cortex, striatum, thalamus, and hypothalamus. Going one step further, in the present study, we also studied maternal differential responses to sad child faces and found activation in the amygdala, anterior cingulate cortex, insula, and hippocampus which have been associated with a variety of processes, including fear processing [35], salience detection [36, 37], and empathizing with the pain of a loved one [38]. One may tentatively suggest that recognizing the own child’s sad face is of salient importance for a mother and activates a threat detection network in the mother [9, 39, 40].

Only few previous studies have presented sad child expressions to mothers [13–16] relying on infant faces. One of these studies [14] found that sad faces of the own child elicit a stronger amygdala response compared to an unknown child. Another line of research has studied maternal responses to sad auditory infant stimuli, i.e., baby cry. In line with our findings, also these studies found maternal activation in the amygdala, anterior cingulate cortex, and insula [32, 33, 41, 42]. Our present study differs from these previous visual and auditory studies in that we used pictures of school-aged children as opposed to infants. Thus, our children were presumably less dependent of their mothers. Nonetheless, we again found strong differential activation in the amygdala, anterior cingulate cortex, and insula indicating the high salience of the own child’s sad face for a mother, which may exist independent of the child’s age. This was further confirmed by our non-significant correlations of child age and peak-voxel BOLD signals in all ROIs. We acknowledge that children’s age ranged between five and twelve years. The correlational analysis does therefore not represent possible alterations in the processing of facial expressions of infants. The question arises whether maternal responses to their own child’s sad affective states alter when they grow up further. To address this issue, it would be desirable to study mothers’ neural responses to children beyond the age of twelve.

One of the challenges in studying maternal neural responses to their child’s affective states is to combine both happy and sad states in one paradigm. For reasons of feasibility, visual studies may primarily have focused on happy facial expressions and auditory studies on infant cry. Sad facial expressions in children and happy auditory stimuli in infants may be more difficult to establish. Relying on school children, the present study was in the position to develop a paradigm that included both happy and sad facial expressions of children. The present paradigm therefore adds to the literature in that it allows studying the direct comparison of neural response to sad and happy child faces to further increase our understanding of affect recognition in healthy mothers.

While sad faces produced differential activations in the amygdala and ACC, happy faces (own versus unfamiliar child) produced activation in the hippocampus and parahippocampus. Although we did not find activation of the ventral striatum as the candidate region for reward processes, these latter regions have also been associated with reward [8, 43, 44] in addition to specific mnestic functions like the retrieval of person knowledge and episodic memories [31, 45–47]. Strathearn and colleagues [13, 14, 17] consistently found activation in the ventral striatum in response to the own infant, in mothers of newborns. It is possible that happy infants have a more direct rewarding character as indicated by activation in the ventral striatum, whereas happy facial expressions of older children do only indirectly elicit reward processes in the mother via maternal memories of the child.

Our behavioral data showed that mothers recognized pictures of happy faces of their own child faster and rated them as more arousing compared to the unfamiliar child. This finding might be due to the perceptual and categorical distinctiveness of happy facial expressions [48, 49]. In comparison to negative faces, the smile of happy expressions is a unique facial feature. This could explain why happy faces were recognized faster which is reported to be a common phenomenon in affect categorization tasks [50]. The question arises whether differences between happy and sad face conditions were confounded by salience differences between conditions. This would not affect our results for sad faces nor the conjunction analysis. However, for the contrast of happy faces of one’s own child compared to sad faces (compared to the unfamiliar child) this could potentially confound the findings. To address this issue, we performed an additional second-level full-factorial analysis including response times and arousal ratings as covariates. This analysis, again showed significant activations in the inferior parietal gyrus and the right parahippocampus (p < .001, uncorrected), and additionally in the inferior frontal gyrus (p < .001, uncorrected) in response to happy faces. This activation pattern is similar to the findings of the initial analysis suggesting that findings in the happy condition are not confounded by better perceptibility of happy faces.

From an evolutionary perspective, it is likely that specific brain mechanisms in mothers have developed to secure the survival of their offspring. Different stages of processing may be distinguished in maternal responses to their child’s affective states, starting from early (subcortical) visual information processing through more complex (cortical/prefrontal) perceptive processes to motor actions. At the same time, it is obvious that adequate maternal responses to sad and happy affective states will differ. Our data from the affect recognition task (requiring perception but not motor action) indicate that this difference is already present during perceptive stages of the maternal responses, even during early subcortical visual information processing. It may be speculated that these early differential responses to sad and happy faces may facilitate fast and adequate reactions of the mothers, e.g., vigilant protective behavior in response to sad child faces and sensitive parenting behavior in response to happy, rewarding child faces. We acknowledge that both, vigilant protective and sensitive maternal behavior might not be easily distinguishable, because they are both in the interest of a child. On a psychological level, both kinds of parenting behavior require a mother’s sense of empathy which the data of the conjunction analysis confirm. However, we acknowledge that we have not directly measured vigilant and sensitive maternal behavior to support this assumption.

In addition to dissociable networks, our paradigm also allowed us to identify brain areas showing overlapping activations to both happy and sad expressions of the own child. This finding is of particular interest, as children begin to report more complex and mixed emotions during school age [19]. We found activation in areas associated with cognitive and affective aspects of empathy, such as the superior temporal gyrus and the insular regions (for a meta-analysis, see [51]). The common network possibly reflects healthy mothers’ ability to understand their child’s intentions and wishes, and to react sensitively to them.

In conclusion, mothers showed dissociable and overlapping activations in response to sad and happy facial expressions of the own child. Sad facial expressions activated a threat detection network (including the amygdala and anterior cingulate cortex). Happy facial expressions of the own child activated a reward network (including inferior frontal gyrus and (para)hippocampus). Common activation of sad and happy facial expressions activated an empathy network (including the insula and superior temporal gyrus). Our data indicate that mothers activate these networks not only in response to infants’, but also to school children’s affective states. Our data also suggest that dissociable responses to sad and happy facial expressions can already be observed during perceptive stages of the maternal responses, even during early subcortical visual information processing. Altered activation of these neural networks might negatively impact parenting behavior. This has to be shown in future studies including mothers-child dyads showing dysfunctional interaction.

## Supporting information

S1 Table Results for the contrast: (sad_oc > happy_oc) > (sad_uc > happy_uc). (DOCX)

S2 Table Results for the contrast: (happy_oc > sad_oc) > (happy_uc > sad_uc). (DOCX)
