# Supplementary material for: Dissociating maternal responses to sad and happy facial expressions of their own child: An fMRI study
Source: PLoS One. 2017 Aug 14;12(8):e0182476. doi: 10.1371/journal.pone.0182476 (PMC5555664; doi:10.1371/journal.pone.0182476)
Supplement: S1 Table — (DOCX) [file pone.0182476.s001.docx]

S1 Table: Results for the contrast: (sad_oc > happy_oc) > (sad_uc > happy_uc)

|  |  |  | **MNI coordinates** | | | |  |
| --- | --- | --- | --- | --- | --- | --- | --- |
| **Region** | **BA** | **R/L** | **x** | **y** | **z** | **T value** | |
| Frontal |  |  |  |  |  |  | |
| Middle orbitofrontal gyrus^a^ | 10 | L | -2 | 46 | -10 | 9.33 | |
| Inferior orbitofrontal gyrus^a^ | 10 | L | -30 | 30 | -18 | 5.22 | |
|  | 47 | R | 24 | 16 | -22 | 5.78 | |
| Anterior cingulate gyrus^a^ | 32 | L | -2 | 42 | -6 | 8.68 | |
|  | 32 | R | 4 | 16 | 22 | 4.95 | |
| Temporal |  |  |  |  |  |  | |
| Middle temporal gyrus^a^ | 21 | L | -58 | -10 | -20 | 8.36 | |
|  | 21 | R | 40 | 20 | -36 | 5.17 | |
| Parietal |  |  |  |  |  |  | |
| Posterior cingulate gyrus^a^ | 31 | L | -4 | -54 | 28 | 9.22 | |
| Precuneus^a^ | 31 | L | -6 | -54 | 26 | 9.02 | |
| Postcentral gyrus^a^ | 3 | R | 40 | -36 | 68 | 5.60 | |
| Occipital |  |  |  |  |  |  | |
| Fusiform gyrus^a^ | 37 | L | -32 | -36 | -18 | 5.51 | |
| Subcortical |  |  |  |  |  |  | |
| Amygdala^a^ |  | L | -28 | -4 | -22 | 4.94 | |
|  |  | R | 26 | -2 | -28 | 4.94 | |
| Hippocampus^a^ |  | L | -26 | -6 | -24 | 4.75 | |
|  |  | R | 26 | -4 | -24 | 4.83 | |
| Parahippocampal gyrus^a^ | 35 | R | 24 | -2 | -28 | 4.99 | |
| Insula^a^ | 47 | L | -28 | 10 | -20 | 3.96 | |
|  | 47 | R | 26 | 14 | -20 | 5.15 | |
| BA: Brodman’s area; R: right, L: left; MNI: Montreal Neurological Institute; ^a^p<.05 (FWE), corrected for whole-brain volume;  ^b^p<.05 (FWE), corrected for small volume (SVC) | | | | | | | |
