# Supplementary material for: Dissociating maternal responses to sad and happy facial expressions of their own child: An fMRI study
Source: PLoS One. 2017 Aug 14;12(8):e0182476. doi: 10.1371/journal.pone.0182476 (PMC5555664; doi:10.1371/journal.pone.0182476)
Supplement: S2 Table — (DOCX) [file pone.0182476.s002.docx]

|  |  |  | **MNI coordinates** | | | | |  |
| --- | --- | --- | --- | --- | --- | --- | --- | --- |
| **Region** | **BA** | **R/L** | **x** | **y** | **z** | **T value** |  |  |
| Frontal |  |  |  |  |  |  |  |  |
| Superior frontal gyrus^a^ | 6 | R | 8 | 28 | 40 | 4.68 |  |  |
| Inferior frontal gyrus^a^ | 13 | R | 40 | 32 | 28 | 3.91 |  |  |
| Supplemental motor area^a^ | 32 | L | 2 | 22 | 46 | 4.36 |  |  |
| Occipital |  |  |  |  |  |  |  |  |
| Lingual gyrus^a^ | 19 | R | 20 | -60 | 2 | 4.33 |  |  |
| BA: Brodman’s area; R: right, L: left; MNI: Montreal Neurological Institute; ^a^p<.05 (FWE), corrected for whole-brain volume;  ^b^p<.05 (FWE), corrected for small volume (SVC); | | | | | | |  |  |

S2 Table: Results for the contrast: (happy_oc > sad_oc) > (happy_uc > sad_uc)
